# Outcomes of Arteriotomy Closure Technique for Carotid Endarterectomy: Bovine Pericardial Patch Closure *versus* Primary Closure

**DOI:** 10.21470/1678-9741-2020-0716

**Published:** 2022

**Authors:** Necip Becit, Fehim Can Sevil, Mehmet Tort, Fahri Adalı

**Affiliations:** 1 Department of Cardiovascular Surgery, Afyonkarahisar Health Sciences University Hospital, Afyonkarahisar, Turkey

**Keywords:** Carotid Artery, Carotid, Stenosis, Endarterectomy, Carotid, Heterografts, Pericardium, Treatment Outcome, Time Factors

## Abstract

**Introduction:**

The aim of our study was to compare the primary closure (PRC) and patch angioplasty closure (PAC) of carotid artery following carotid endarterectomy (CEA).

**Methods:**

Data of patients who underwent CEA in the period from January 2005 to June 2020 were reviewed through files. Demographic characteristics, information about the operation, and postoperative follow-up outcomes of the patients were compared.

**Results:**

Of the 144 CEA cases included in the study, PRC and PAC were applied to 62 (43.7%) and 82 (56.3%) patients, respectively, for the carotid artery closure. Duration of surgery and carotid artery clamping time were not different between the PRC and PAC groups (106.73±17.13 minutes *vs.* 110.48±20.67 minutes, *P*=0.635; 24.25±11.56 minutes *vs.* 25.19±8.99 minutes, *P*=0.351, respectively). Postoperative respiratory impairment was more common in the PRC group (*P*=0.012); however, nerve injuries (*P*=0.254), surgical wound hematomas (*P*=0.605), surgical site infections (*P*=0.679), and mortality (*P*=0.812) were not significantly different between the groups. During the mean patient follow-up time of 26.13±19.32 months, restenosis was more common in the PRC group than in the PAC group (n=26, 41.9% *vs.* n=4, 4.9%, respectively; *P*=0.003). Frequencies of stroke (n=4, 2.8% *vs.* n=2, 2.4%, respectively; *P*=0.679), transient ischemic attacks (n=2, 1.4% *vs.* n=0, 0%, respectively; *P*=0.431), and mortality (n=4, 6.5% *vs.* n=4, 4.9%, respectively; *P*=0.580) were not significantly different between the PRC and PAC groups.

**Conclusion:**

We are of the opinion that the PAC method is effective and safe for carotid artery closure in patients undergoing CEA.

**Table:** 

Abbreviations, acronyms & symbols	
CCA	= Common carotid artery
CEA	= Carotid endarterectomy
ICA	= Internal carotid artery
PAC	= Patch angioplasty closure
PRC	= Primary closure
PTFE	= Polytetrafluoroethylene
TIA	= Transient ischemic attack

## INTRODUCTION

Although carotid endarterectomy (CEA) is the gold standard treatment in carotid artery stenosis, the selection of the method for closure of the carotid artery is still controversial, along with plaque removal techniques, use of shunts, and the method of anesthesia, remaining a matter of debate^[1,2]^. Primary closure (PRC) and patch angioplasty closure (PAC) of the carotid artery or eversion techniques are the options^[3,4]^. PAC with autologous saphenous vein is associated with excellent outcomes in the early period; however, complications including ballooning or ruptures in the saphenous vein caused a search for different materials to be used in PAC^[5]^. Despite the ease of use of prosthetic patches, the increased risk of infection leading to bleeding complications and thrombosis is more associated with prosthetic patches compared to autogenous tissue patches^[6]^. It has been reported that infections, thrombogenicity, and bleeding from the suture line occur less frequently, but the tissue compatibility is favorable in association with the use of bovine pericardium patches for PAC^[7]^. Restenosis associated with intimal hyperplasia and underlying atherosclerosis can occur after CEA^[8]^. Restenosis has been defined as > 50% occlusion in the carotid artery. The incidence of restenosis has been reported in the range from 7% to 36%^[8-12]^. Some studies have defined restenosis as an obstruction of > 70% in the carotid artery and reported that there are no differences between PRC and PAC in terms of the occurrence of restenosis^[12-14]^. However, most studies demonstrate the superiority of the PAC method in association with low rates of restenosis development, bleeding complications, and infections, and recommend PAC for closure of the carotid artery^[4,15,16]^.

The aims of our study are to determine the rates of postoperative restenosis, stroke, or transient ischemic attacks (TIA) and other complications in patients undergoing CEA and to compare these results between the PAC with bovine pericardium and PRC groups.

## METHODS

This study was approved by the Ethics Committee of Afyonkarahisar Health Sciences University (2011-KAEK-2) and the requirement for informed consent was waived by the committee.

### Patient Selection

This study included patients who underwent CEA under regional anesthesia in our clinic in the period from January 2005 to June 2020. Neurological symptoms were defined as hemispheric infarctions, TIA, retinal infarctions, and retinal TIA associated with carotid artery stenosis. Asymptomatic patients were defined as individuals with carotid artery stenosis but having no neurological complaints. CEA was performed in symptomatic patients with 50-99% stenosis and in asymptomatic patients with 70-99% stenosis. Patients who underwent CEA under general anesthesia, who underwent carotid artery surgery previously, who underwent bilateral CEA, and who underwent PAC with a prosthetic patch other than bovine pericardium were excluded from the study.

### Preoperative Care and Diagnostic Workup

A detailed anamnesis was obtained from the study patients. Each patient included in the study underwent a neurological examination. The patients underwent relevant physical examinations and laboratory tests for the detection of comorbidities. Firstly, a duplex ultrasound was used for examining the carotid arteries. Flow velocities in the common carotid artery and the internal carotid artery (ICA) were measured. The degree of stenosis in these arteries was determined using the North American Symptomatic Carotid Endarterectomy Trial criteria.

A computed tomography angiography scan was performed for preoperative planning and for the confirmation of findings obtained from the duplex ultrasound imaging.

### Surgical Technique

After transferring the patient to the recovery room, full monitoring was applied. Cervical plexus block was performed under Doppler ultrasound guidance. A 100 IU/kg dose of unfractionated heparin was administered to the patients intravenously. Prior to CEA clamping for five minutes, we performed the awake test, which included speech, grasping a rubber ball, and toe flexion and extension. After clamping, we performed the awake test immediately and every five minutes thereafter until the cross-clamp is removed. An arteriotomy extending from the common carotid artery to the ICA was performed. If abnormal findings on the awake test were noted during the operation, we inserted a carotid artery shunt to allow patients to recover from the cerebral ischemic state. When placing a shunt, the part of the shunt that would remain in the ICA was placed first. Then, the part of the shunt that would remain in the common carotid artery was placed. Using a Freer Elevator, the space between the vascular endothelium and the plaque was entered. The plaque was dissected from the blood vessel lumen, and the lumen was cleaned with a solution containing unfractionated heparin until the interior lining of the lumen became smooth. In all patients, the shunt was removed before the complete closure of the anastomosis. After the evacuation of the air, the anastomosis was completed. The choice of CEA technique during each operation was based on the clinical judgment and discretion of the attending vascular surgeon because of technical concerns or physician preference. Patients with ICA diameters < 5 mm were relegated to a PAC category. If abnormal findings on the awake test were noted during the operation, we performed PRC technic and tried to prevent loss of time. Similarly, PAC was not performed in patients with ICA diameters > 5 mm who reported chest pain, shortness of breath, and who had changes such as tachycardia and arrhythmia in the electrocardiogram; PRC technique was preferred. We preferred the PAC technic in patients with high risk of tortuosity of carotid artery after carotid artery endarterectomy. Although operative technique was not randomized, both groups were otherwise sufficiently similar in number and clinical characteristics for valid statistical comparison.

All surgeons used bovine pericardium for patching, and all patients were transferred to the intensive care unit after surgery and were fully monitored. Detailed neurological examinations of the patients were performed. Blood samples were collected from the patients to perform laboratory tests. Patients with visual analog scores > 6 received pain medications. Antihypertensive therapy was started to the patients with blood pressure values > 140 mmHg. Patients with stable vital signs were transferred to the inpatient unit when no complications were detected.

Patients with coronary artery disease comorbid with carotid artery stenosis, who needed to undergo coronary artery bypass surgery, underwent coronary artery bypass surgery on the first postoperative day after CEA. Patients with peripheral arterial disease comorbid with carotid artery stenosis, who needed surgical intervention, underwent surgery for peripheral arterial disease at the end of the first postoperative month after CEA.

### Data Collection and Follow-up

Demographic characteristics, findings from preoperative ultrasonography and radiological imaging tests, operative information, and postoperative and follow-up outcomes of the patients were retrospectively retrieved and analyzed. Postoperative follow-up visits with three-month intervals were scheduled for the patients. At the follow-up visits, the patients underwent neurological examinations and ultrasound imaging to detect potential restenosis.

### Statistical Analysis

Statistical analyses were performed using IBM Corp. Released 2011, IBM SPSS Statistics for Windows, version 20.0, Armonk, NY: IBM Corp. Fisher’s exact test was used for comparing categorical variables. One-way analysis of variance was used for the comparison of continuous variables. A two-tailed probability value of < 0.05 was considered significant. All values were presented as mean ± standard deviation or numbers (%), unless stated otherwise.

## RESULTS

The mean age of the patients who underwent CEA was 70.06±8.71 years and was not different between the PRC and PAC groups (70.32±9.29 *vs*. 69.85±8.35, *P*=0.833, respectively). Of the patients, 116 (80.6%) were men. There were no significant differences in gender between the groups (P=0.342).

Arteriotomies were closed by PRC in 62 (43.7%) patients and by PAC in 82 (56.3%) patients. The most common presenting complaint of the patients was a hemispheric infarction, which was present in 78 (54.2%) patients. The most common comorbid disease was hypertension in the patients with coronary artery stenosis. Hypertension was present in 104 (72.2%) patients. Demographic characteristics, comorbid diseases, history of smoking, and presenting symptoms at admission were not different between the groups. These findings are presented in Table 1.

**Table 1:** Baseline characteristics of study population stratified according to carotid endarterectomy technique.

Characteristic	Total (n=144)	PRC (n=62)	PAC (n=82)	*P*-value
Age (years)	70.06±8.71	70.32±9.29	69.85±8.35	0.833
Male	116 (80.6)	48 (77.4)	68 (82.9)	0.342
Comorbidity				
Hypertension	104 (72.2)	44 (71)	60 (73.2)	0.836
Coronary artery disease	94 (65.3)	42 (67.7)	52 (63.4)	0.703
Diabetes mellitus	68 (47.2)	22 (35.5)	46 (56.1)	0.083
Angina	60 (41.7)	22 (35.5)	38 (46.3)	0.355
Hyperlipidemia	50 (34.7)	18 (29)	32 (39)	0.378
Chronic obstructive pulmonary disease	30 (20.8)	12 (19.4)	18 (22)	0.788
Myocardial infarction	22 (15.5)	14 (22.6)	8 (10)	0.192
Peripheral arterial disease	20 (13.9)	12 (19.4)	8 (9.8)	0.310
Chronic kidney disease	12 (8.3)	6 (9.7)	6 (7.3)	0.521
Current or ex-smoker	42 (29.2)	22 (35.5)	20 (24.4)	0.305
Neurological symptoms				
Asymptomatic	26 (18.1)	10 (16.1)	16 (19.5)	0.712
Symptomatic				
Hemispheric infarct	78 (54.2)	38 (61.3)	40 (48.8)	0.291
Hemispheric TIA	30 (20.8)	14 (22.6)	16 (19.5)	0.751
Retinal infarct	4 (2.8)	0 (0)	4 (4.9)	0.503
Retinal TIA	6 (4.2)	0 (0)	6 (7.3)	0.254

Values are presented as mean ± standard deviation or number (%)

*P*-values calculated by Pearson’s chi-squared and Fisher’s exact tests

PAC=patch angioplasty closure; PRC=primary closure; TIA=transient ischemic attack

Blood flow velocity in the common carotid artery was 52.74±15.51 cm/sec as measured by Doppler ultrasonography. There were no differences in the common carotid artery blood flow velocities between the groups (PRC 49.58±15.88 cm/sec *vs*. PAC 55.25±14.94 cm/sec, *P*=0.109). Mean blood flow velocity in the internal carotid artery was 139.75±40.09 cm/sec and it was not different between the groups (PRC 140.39±48.79 cm/sec *vs*. PAC 139.27±32.67 cm/sec, *P*=0.341). Sixteen (11.1%) patients were found to have a 50-69% stenosis in the carotid artery in the Doppler ultrasonography examinations *vs*. six patients (4.2%) who were found to have a 50-69% stenosis by the computed tomography angiography. In the computed tomography angiography, 94 (65.3%) patients were found to have > 50% stenosis in the contralateral carotid artery; however, there was not a significant difference between the PRC and PAC groups (n=40, 64.5% vs. n=54, 65.9%, *P*=0.906). The data about the carotid artery imaging findings of the patients are presented in Table 2.

**Table 2:** Characteristics of carotid arteries according to carotid endarterectomy (CEA) technique.

Characteristic	Total (n=144)	PRC (n=62)	PAC (n=82)	*P*-value
CEA side				
Peak systolic velocity of CCA on Doppler ultrasound	52.74±15.51	49.58±15.88	55.25±14.94	0.109
Peak systolic velocity of ICA on Doppler ultrasound	139.75±40.09	140.39±48.79	139.27±32.67	0.341
Carotid artery stenosis on Doppler ultrasound				
50-69%	16 (11.1)	6 (9.7)	10 (12.2)	0.522
> 70%	128 (88.9)	76 (90.3)	72 (87.8)	0.485
Carotid artery stenosis on computed tomography				
50-69%	6 (4.2)	2 (3.2)	4 (4.9)	0.605
> 70%	138 (95.8)	60 (96.8)	78 (95.1)	0.545
Contralateral carotid stenosis on computed tomography				
< 50%	50 (34.7)	22 (35.5)	28 (34.1)	0.785
≥ 50	94 (65.3)	40 (64.5)	54 (65.9)	0.906

Values are presented as mean ± standard deviation or number (%)

CCA=common carotid artery; ICA=internal carotid artery; PAC=patch angioplasty closure; PRC=primary closure

*P*-values calculated by Pearson’s chi-squared and Fisher’s exact tests

CEA was performed in the right carotid artery in 72 (50%) cases. Bilateral CEA was not performed in any patient. The side of CEA was not different between the groups (right-sided CEA, PRC n=34, 54.8% *vs*. PAC n=38, 46.3%, *P*=0.510). Duration of surgery was shorter in the PRC group than in the PAC group, but the difference was not significant (106.73±17.13 minutes *vs*. 110.48±20.67 minutes, *P*=0.635, respectively). Duration of the carotid artery clamping time was shorter in the PRC group than in the PAC group, but the difference was not significant (24.25±11.56 minutes *vs*. 25.19±8.99 minutes, *P*=0.351, respectively). Shunts were placed when abnormal findings were obtained in the awake test after regional anesthesia. All of these patients were in the PAC group (*P*=0.033). Postoperative respiratory impairments were observed in ten (13.1%) patients in the PRC group, but no such problems were observed in the PAC group (*P*=0.012). Nerve injuries occurred in six (4.2%) patients, wound site hematomas occurred in six patients, infections at the incision site occurred in four (2.8%) patients, and early mortality occurred in four (2.8%) patients. There were no significant differences in these untoward events between the groups. Analgesic medications were given to 18 (12.5%) patients who scored > 6 points in the follow-up period in the intensive care unit. The need for analgesic treatment was more common in the PAC group (*P*=0.039). Eighteen (12.5%) patients with hypertension were given antihypertensive medication for blood pressure stabilization in the intensive care unit. Antihypertensive drugs were used more frequently in the PAC group (n=14, 17.1%), but there was not a significant difference between the two groups (*P*=0.162). Eight (5.6%) patients who underwent CEA received blood transfusions, but there was not a significant difference between the PRC group and the PAC group (n=6, 9.7% *vs*. n=2, 2.4%, *P*=0.308, respectively). The length of stay in the intensive care unit was shorter in the PAC group (*P*=0.011). The length of stay at the hospital was shorter in the PAC group, but this difference was not significant (*P*=0.956). The operative and postoperative data of the patients are presented in Table 3.

**Table 3:** Operative details and outcomes of carotid endarterectomy (CEA).

Variable	Total (n=144)	PRC (n=62)	PAC (n=82)	*P*-value
CEA side				
Right-sided CEA	72 (50)	34 (54.8)	38 (46.3)	0.510
Left-sided CEA	72 (50)	28 (45.2)	44 (53.7)	0.634
Operative time (min)	108.34±18.69	106.73±17.13	110.48±20.67	0.635
Clamping time (min)	24.79±10.11	24.25±11.56	25.19±8.99	0.351
Usage of shunt	12 (8.3)	0 (0)	12 (14.6)	0.033
Complications				
Respiratory	10 (6.9)	10 (16.1)	0	0.012
Nerve injury	6 (4.2)	0	6 (7.3)	0.254
Hematoma	6 (4.2)	2 (3.2)	4 (4.9)	0.605
Wound infection	4 (2.8)	2 (3.2)	2 (2.4)	0.679
In-hospital mortality	4 (2.8)	4 (6.5)	0	0.182
Use of intravenous painkiller	18 (12.5)	2 (3.2)	16 (19.5)	0.039
Use of intravenous blood pressure control drug	18 (12.5)	4 (6.5)	14 (17.1)	0.162
Blood transfusion	8 (5.6)	6 (9.7)	2 (2.4)	0.308
Intensive care unit stay (days)	1.53±3.41	2.16±5.18	1.05±0.21	0.011
In-hospital stay (days)	3.58±3.19	4.40±4.7	2.98±0.96	0.956

Values are presented as mean ± standard deviation or number (%)

*P*-values calculated by Pearson’s chi-squared and Fisher’s exact tests

PAC=patch angioplasty closure; PRC=primary closure

The mean follow-up period of the patients was 26.13±19.32 months. At the follow-up visits scheduled at three-month intervals, the patients underwent examinations to detect potential restenosis, stroke, and TIA. Mortality rates in the follow-up period were evaluated. Recurrent stenosis of > 30% in the carotid artery was identified in 30 (20.8%) patients who underwent CEA. Restenosis was more common in the PRC group than in the PAC group (n=26, 41.9% *vs*. n=4, 4.9% respectively; *P*=0.003). Stroke, TIA, and mortality occurred in four (2.8%), two (1.4%), and eight (5.6%) patients, respectively, but there were no differences between the groups. The postoperative stroke in two patients occurred due to contralateral carotid artery stenosis. Mortality occurred in the patients due to coronary artery disease. The follow-up data of the patients are presented in Table 4. No complications occurred in the treatment and follow-up periods of patients who underwent coronary artery bypass surgery or surgery for peripheral vascular disease after CEA.

**Table 4:** Outcomes of postoperative follow-up.

Variable	Total (n=144)	PRC (n=62)	PAC (n=82)	*P*-value
Carotid artery restenosis				
≥ 30%	30 (20.8)	26 (41.9)	4 (4.9)	0.003
Stroke	4 (2.8)	2 (2.4)	2 (3.2)	0.679
TIA	2 (1.4)	2 (3.2)	0	0.431
Mortality	8 (5.6)	4 (6.5)	4 (4.9)	0.580

Values are presented as mean ± standard deviation or number (%)

*P*-values calculated by Pearson’s chi-squared and Fisher’s exact tests

PAC=patch angioplasty closure; PRC=primary closure; TIA=transient ischemic attack

## DISCUSSION

PRC, PAC, and eversion techniques can be used for arteriotomy closure after CEA^[4]^. Saphenous vein as autologous grafts and Dacron and polytetrafluoroethylene (PTFE) as prosthetic patch materials are preferred to be used in the PAC method^[6,17,18]^. The use of the saphenous vein PAC has decreased due to the need for a second incision and the catastrophic consequences of developing aneurysms or ruptures in the saphenous vein. In Dacron and PTFE prosthetic patches, several complications may occur including susceptibility to infections and bleeding in the suture line^[5,17-20]^. The use of bovine pericardium as the patch material has been demonstrated to be superior over the previously mentioned methods because of high tissue compatibility and low risks of infections and bleeding in the suture line^[6,7]^.

We compared the operative information and postoperative and follow-up outcomes of the patients who underwent CEA between the PRC and PAC groups. We observed several achievements in our study. Firstly, the operative time (*P*=0.635) and the duration of the carotid artery clamping time (*P*=0.351) were longer in the PAC group, but the differences were not statistically significant between the groups. Secondly, all patients with postoperative respiratory impairments underwent carotid artery closure through the PRC method (*P*=0.012). Complications including nerve injuries (*P*=0.254), surgical wound hematomas (*P*=0.605), surgical site infections (*P*=0.679), and mortality (*P*=0.812) were not significantly more common in the PAC group. Thirdly, postoperative restenosis was more common in the PRC group (*P*=0.003). There was not a difference in the occurrence of stroke (*P*=0.679), TIA (*P*=0.431), and mortality (*P*=0.580) between the groups.

An increase in the complications of CEA is observed as the operative time and carotid clamping time prolongs^[11,21,22]^. Although there are publications reporting that the operative time and clamping time are shorter with the PRC method, a significant difference was not observed between the groups in our study^[11]^. We are of the opinion that the results we obtained in our study occurred because of the bovine pericardium’s suitable structure for anastomosis and our surgical experience enabling us to perform PAC swiftly.

Postoperative respiratory impairments were observed in 6.9% of the patients, and all of these patients were in the PRC group (*P*=0.012). We are of the opinion that the higher rate of respiratory problems in the PRC group compared to the PAC group is the result of closing the carotid artery with the PRC method in patients with chest pain, shortness of breath, and confusion during the operation. Nerve injuries after CEA were seen at a rate of 4.2%, and this rate is consistent with the literature^[11]^. Nerve injuries occurred only in the PAC group. We are of the opinion that the nerve injuries occurred due to the traction applied during PAC. Of the patients developing nerve injuries, recurrent laryngeal nerve injury occurred in two patients, and hypoglossal nerve injury occurred in four patients. Full recovery was achieved in these patients without any sequelae during the six-month follow-up period. There were no differences between the groups in terms of hematomas, wound infections, and mortality. It was observed that PAC with bovine pericardium did not cause an increase in postoperative complications.

Patients in the PAC group stayed at the intensive care unit for a shorter time (*P*=0.011) and required more analgesic drugs. We think that the higher need for analgesics occurred because of the traction applied during PAC. Although previous studies reported more blood transfusions in patients undergoing PAC^[11]^, the PRC group in our study received more blood transfusions compared to the PAC group. However, the difference between the groups was not significant. We think that blood transfusions were needed less in the PAC group because bovine pericardium did not cause bleeding in the suture line.

Restenosis can occur following CEA resulting from intimal hyperplasia in the carotid artery and the progression of atherosclerotic disease^[8]^. While the native vessel lumen diameter is increased with PAC procedure, a decrease in vessel lumen diameter is observed because of the depletion of the vessel wall required for the suture line in PRC. As a result, we think that even if hyperplasia and atherosclerosis progress at the same rate in both groups, the rate of restenosis will be lower in the PAC group. Some studies have defined restenosis as a recurrent obstruction of > 50% in the carotid artery, and others have defined it as an obstruction of > 70%. The rates of restenosis have been reported ranging from 7% to 36%^[8-12]^. Despite some studies reporting that PAC is an ineffective method in preventing the development of restenosis and that the outcomes are not different from those of PRC^[11]^, it is widely accepted that the PAC method is superior over PRC in terms of risk of restenosis development^[3,6,8,23]^. In our study, restenosis was defined as > 30% stenosis and impaired luminal blood flow. We preferred this definition because we think that, whether in the PRC group or in the PAC group, even lower degrees of stenoses significantly affect luminal flow due to the deterioration of the carotid artery lumen structure, leading to likelihood of thrombosis. In our study, restenosis occurred less commonly in the PAC group during the follow-up period (*P*=0.003). We are of the opinion that the rationale for the selection of the PRC method in CEA surgery needs to be standardized, including the parameters carotid artery diameter, flow velocity, and intimal wall thickness. Considering our study results, we think that the closure of arteriotomies following CEA surgery should be performed through the PAC method, using bovine pericardium particularly. The absence of differences in the frequencies of stroke, TIA, and mortality between the PRC group and the PAC group in the follow-up period demonstrated that the PAC method did not increase complication rates.

In our study, it was observed that the use of the PAC method for the closure of arteriotomy following CEA surgery did not prolong the operative time and the carotid artery clamping time and that it reduced the length of stay in intensive care, bleeding complications, and most importantly, it caused less postoperative restenosis and did not increase the occurrence of stroke, TIA, and mortality in the follow-up period.

## CONCLUSION

We are of the opinion that the PAC method is effective and safe for the closure of arteriotomies following CEA surgery.

## References

[1] Saraç A (2020). Carotid endarterectomy under local anesthesia: an institutional
report of experience. Turkish J Vasc Surg.

[2] Sevil FC (2020). Management and outcomes of vascular reconstruction in carotid
body tumor resection: retrospective analysis of 60 cases. Eur Arch Otorhinolaryngol.

[3] Nana P, Spanos K, Piffaretti G, Koncar I, Kouvelos G, Zlatanovic P (2020). Long-term durability and safety of carotid endarterectomy closure
techniques. World J Surg.

[4] Naylor AR, Ricco JB, de Borst GJ, Debus S, de Haro J, Halliday A (2018). Editor's choice - management of atherosclerotic carotid and
vertebral artery disease: 2017 clinical practice guidelines of the European
society for vascular surgery (ESVS). Eur J Vasc Endovasc Surg.

[5] O'Hara PJ, Hertzer NR, Krajewski LP, Beven EG (1992). Saphenous vein patch rupture after carotid
endarterectomy. J Vasc Surg.

[6] Ho KJ, Nguyen LL, Menard MT (2012). Intermediate-term outcome of carotid endarterectomy with bovine
pericardial patch closure compared with dacron patch and primary
closure. J Vasc Surg.

[7] Marien BJ, Raffetto JD, Seidman CS, LaMorte WW, Menzoian JO (2002). Bovine pericardium vs dacron for patch angioplasty after carotid
endarterectomy: a prospective randomized study. Arch Surg.

[8] Goodney PP, Nolan BW, Eldrup-Jorgensen J, Likosky DS, Cronenwett JL, Vascular Study Group of Northern New England (2010). Restenosis after carotid endarterectomy in a multicenter regional
registry. J Vasc Surg.

[9] Huizing E, Vos CG, van den Akker PJ, Schreve MA, de Borst GJ, Ünlü Ç (2019). A systematic review of patch angioplasty versus primary closure
for carotid endarterectomy. J Vasc Surg.

[10] Arquizan C, Trinquart L, Touboul PJ, Long A, Feasson S, Terriat B (2011). Restenosis is more frequent after carotid stenting than after
endarterectomy: the EVA-3S study. Stroke.

[11] Avgerinos ED, Chaer RA, Naddaf A, El-Shazly OM, Marone L, Makaroun MS (2016). Primary closure after carotid endarterectomy is not inferior to
other closure techniques. J Vasc Surg.

[12] Avgerinos ED, Go C, Ling J, Naddaf A, Steinmetz A, Abou Ali AN (2016). Carotid artery disease progression and related neurologic events
after carotid endarterectomy. J Vasc Surg.

[13] Demirel S, Attigah N, Bruijnen H, Ringleb P, Eckstein HH, Fraedrich G (2012). Multicenter experience on eversion versus conventional carotid
endarterectomy in symptomatic carotid artery stenosis: observations from the
stent-protected angioplasty versus carotid endarterectomy (SPACE-1)
trial. Stroke.

[14] Lal BK, Beach KW, Roubin GS, Lutsep HL, Moore WS, Malas MB (2012). Restenosis after carotid artery stenting and endarterectomy: a
secondary analysis of CREST, a randomised controlled trial. Lancet Neurol.

[15] Archie JP Jr (2000). A fifteen-year experience with carotid endarterectomy after a
formal operative protocol requiring highly frequent patch
angioplasty. J Vasc Surg.

[16] Bond R, Rerkasem K, Naylor AR, Aburahma AF, Rothwell PM (2004). Systematic review of randomized controlled trials of patch
angioplasty versus primary closure and different types of patch materials
during carotid endarterectomy. J Vasc Surg.

[17] AbuRahma AF, Hopkins ES, Robinson PA, Deel JT, Agarwal S (2003). Prospective randomized trial of carotid endarterectomy with
polytetrafluoroethylene versus collagen-impregnated dacron (Hemashield)
patching: late follow-up. Ann Surg.

[18] AbuRahma AF, Hannay RS, Khan JH, Robinson PA, Hudson JK, Davis EA (2002). Prospective randomized study of carotid endarterectomy with
polytetrafluoroethylene versus collagen-impregnated Dacron (Hemashield)
patching: perioperative (30-day) results. J Vasc Surg.

[19] Archie JP Jr, Green JJ Jr. (1990). Saphenous vein rupture pressure, rupture stress, and carotid
endarterectomy vein patch reconstruction. Surgery.

[20] McCready RA, Siderys H, Pittman JN, Herod GT, Halbrook HG, Fehrenbacher JW (1992). Delayed postoperative bleeding from polytetrafluoroethylene
carotid artery patches. J Vasc Surg.

[21] Kim JW, Huh U, Song S, Sung SM, Hong JM, Cho A (2019). Outcomes of carotid endarterectomy according to the anesthetic
method: general versus regional anesthesia. Korean J Thorac Cardiovasc Surg.

[22] Kim JH, Cho YP, Kwon TW, Kim H, Kim GE (2012). Ten-year comparative analysis of bovine pericardium and
autogenous vein for patch angioplasty in patients undergoing carotid
endarterectomy. Ann Vasc Surg.

[23] Huizing E, Vos CG, Hulsebos RG, van den Akker PJ, Borst GJ, Ünlü Ç (2018). Patch angioplasty or primary closure following carotid
endarterectomy for symptomatic carotid artery stenosis. Surg J (N Y).

